# Negative transcriptional control of ERBB2 gene by MBP-1 and HDAC1: diagnostic implications in breast cancer

**DOI:** 10.1186/1471-2407-13-81

**Published:** 2013-02-19

**Authors:** Flavia Contino, Claudia Mazzarella, Arianna Ferro, Mariavera Lo Presti, Elena Roz, Carmelo Lupo, Giovanni Perconti, Agata Giallongo, Salvatore Feo

**Affiliations:** 1 Dipartimento di Scienze e Tecnologie Molecolari e Biomolecolari, Università di Palermo, Viale delle Scienze, Ed. 16, Palermo I-90128, Italy; 2 Istituto di Biomedicina e Immunologia Molecolare, CNR, Via Ugo La Malfa, 153, Palermo I-90146, Italy; 3 Unità di Anatomia Patologica, Dipartimento Oncologico di III livello La Maddalena, Palermo, Italy

**Keywords:** MBP-1, ERBB2, Transcriptional regulation, Histone Deacetylase, Breast cancer

## Abstract

**Background:**

The human ERBB2 gene is frequently amplified in breast tumors, and its high expression is associated with poor prognosis. We previously reported a significant inverse correlation between Myc promoter-binding protein-1 (MBP-1) and ERBB2 expression in primary breast invasive ductal carcinoma (IDC). MBP-1 is a transcriptional repressor of the c-MYC gene that acts by binding to the P2 promoter; only one other direct target of MBP-1, the COX2 gene, has been identified so far.

**Methods:**

To gain new insights into the functional relationship linking MBP-1 and ERBB2 in breast cancer, we have investigated the effects of MBP-1 expression on endogenous ERBB2 transcript and protein levels, as well as on transcription promoter activity, by transient-transfection of SKBr3 cells. Reporter gene and chromatin immunoprecipitation assays were used to dissect the ERBB2 promoter and identify functional MBP-1 target sequences. We also investigated the relative expression of MBP-1 and HDAC1 in IDC and normal breast tissues by immunoblot analysis and immunohistochemistry.

**Results:**

Transfection experiments and chromatin immunoprecipitation assays in SKBr3 cells indicated that MBP-1 negatively regulates the ERBB2 gene by binding to a genomic region between nucleotide −514 and −262 of the proximal promoter; consistent with this, a concomitant recruitment of HDAC1 and loss of acetylated histone H4 was observed. In addition, we found high expression of MBP-1 and HDAC1 in normal tissues and a statistically significant inverse correlation with ErbB2 expression in the paired tumor samples.

**Conclusions:**

Altogether, our in vitro and in vivo data indicate that the ERBB2 gene is a novel MBP-1 target, and immunohistochemistry analysis of primary tumors suggests that the concomitant high expression of MBP-1 and HDAC1 may be considered a diagnostic marker of cancer progression for breast IDC.

## Background

The ERBB2 (Her2/Neu) gene encodes a tyrosine kinase receptor whose abnormal activity is linked to oncogenesis in breast cancer. In fact, ERBB2 gene amplification is found in 20−30% of primary breast tumors, and it is usually associated with poor clinical prognosis. In these tumors, ErbB2 receptor overexpression activates several intracellular signalling pathways, such as the Ras/Erk and PI3K/AKT pathways [1], whose effects on c-MYC oncogene transcription and Myc protein stability have been demonstrated [2]. The treatment of ERBB2-amplified breast tumor cells with the ErbB2-specific antibody trastuzumab causes cell cycle arrest accompanied by a decrease in PI3K/Akt activity and the downregulation of c-MYC and D-type cyclins; on the other hand, ectopic expression of c-MYC in ERBB2-overexpressing SKBr3 cells partially rescues the cells from functional ERBB2 inactivation [3,4]. Several studies have reinforced the significance of c-MYC as an ERBB2 effector and the functional role that the two genes play in breast cancer progression (for a review, see [5]).

The c-MYC gene is regulated at multiple levels. One of the regulators, the Myc promoter-binding protein-1 (MBP-1), was originally identified in HeLa cells as a transcriptional repressor which binds to the human c-MYC P2 promoter, negatively affecting transcription. This factor competes for the TATA-binding protein (TBP) and prevents the formation of the transcription initiation complex [6,7]. MBP-1 is a short form of the 48 kDa alpha-enolase protein, lacking the first 96 amino acid. Several studies support the existence of a single ENO1 gene transcript from which both alpha-enolase and MBP-1 arise through the use of alternative translation initiation sites [8,9]. More recently, it has been reported that a shorter variant transcript, originating from intron III of the ENO1 gene, may contribute to MBP-1 expression in a variety of normal tissues and cancer cells [10]. Exogenous MBP-1 expression inhibits the growth of breast tumors in nude mice [11], induces cell death in neuroblastoma cells [12], suppresses proliferation in non-small-cell lung cancer cells [13], and induces G0–G1 growth arrest in chronic myeloid leukemia cells [14]. Moreover, a role for MBP-1 in tumor invasion and metastasis has been proposed for follicular thyroid carcinoma and gastric cancer [15,16]. MBP-1 may exert its function as a single factor, in concert with other factors, or through physical interaction with its identified cellular partners: MIP-2/sedlin [17], histone deacetylase 1 (HDAC1) [18], the kelch protein NS1-BP [19], and the Notch 1 receptor intracellular domain [20]. Besides c-MYC, only one other direct target of MBP-1, the COX2 gene, has been identified so far [16].

Consistent with its negative regulatory role on cell growth, the endogenous level of MBP-1 in tumor cells is low; in MCF-7 breast cancer cells, glucose concentration and hypoxia have been reported to modulate MBP-1 expression and its binding to the c-MYC promoter, consequently affecting cell proliferation [21,22]. Thus, MBP-1 appears to be one of the factors controlling cell growth and proliferation, and alterations in its expression level induced by the tumor microenvironment may contribute to cancer development.

Our previous studies have indicated that MBP-1 is expressed and easily detectable in normal breast epithelial cells, but a loss of expression occurs in most primary invasive ductal carcinomas (IDC) of the breast. Furthermore, MBP-1 expression inversely correlates with expression levels of the ErbB2 and Ki67 proteins [23]. On the basis of these observations, we hypothesized a direct functional link between MBP-1 and the ERBB2 gene in human breast carcinomas.

In the present study, we provide evidence that MBP-1 inhibits the expression of the ERBB2 gene in SKBr3 breast cancer cells by interacting with the promoter region. In addition, we show that HDAC1 is recruited to the same region of the ERBB2 promoter which is bound by MBP-1. Finally, we report a significant correlation between MBP-1, HDAC1 and ERBB2 protein expression in primary breast carcinomas. Taken together, our findings indicate that the ERBB2 gene is a target of MBP-1 and suggest that the concomitant high expression of MBP-1 and HDAC1 may be considered a diagnostic marker for IDC.

## Methods

### Cell culture and tumor tissues

The ERBB2-amplified human breast cancer cell line SKBr3, was purchased from American Type Culture Collection (ATCC, Rockville, MD). Cells were cultured in DMEM medium supplemented with 10% fetal bovine serum, 2 mM glutamine and 100 μg/ml penicillin/streptomycin (Invitrogen, Carlsbad, CA).

Tumor tissue samples were from 45 patients submitted to routine histopathological examination at the Anatomic Pathology Unit of La Maddalena Hospital in Palermo. All experiments using human tissues were performed with the written patients’ informed consent and with the approval of Institutional Review Boards from La Maddalena Hospital.

### Reporter and effector plasmid constructs

The construction of the effector plasmid pFlag-MBP-1 has been described previously [19]. For the reporter constructs, the relevant regions of the ERBB2 promoter, including 44 base pairs (bp) of the first exon, were obtained by PCR amplification of genomic DNA from a human-mouse hybrid cell line containing only chromosome 17 [24]. Three DNA fragments, spanning 306-, 558- and 787-bp, were amplified with primers containing restriction sites and cloned into the luciferace vector pGL3-basic (Promega, Madison, WI). In order to confirm the nucleotide sequence and the correct orientation of the cloned fragments, the three reporter plasmids, pG-E300, pG-E500 and pG-E700 were subjected to cycle-sequencing on an ABI 3130 genomic analyzer, according to the manufacturer’s instructions (Applied Biosystems, Foster City, CA),

### Cell transfection and luciferase reporter assay

SKBr3 cells were transfected with Lipofectamine LTX reagent in OptiMem medium as instructed by the manufacturer (Invitrogen). For RT-PCR, western blot and ChIP analyses 1.5x10^6^ cells in 10 mm culture dishes were transfected with either the pFlag-MBP1 (3.5 or 7.5 μg) or pFlag-CMV plasmid (7.5 μg) and cell extracts were prepared 48 hrs after transfection. An aliquot of the transfected cells was routinely monitored for transfection efficiency by immunofluorescence assay and Western blot analysis with anti-Flag antibodies. Only samples yielding more than 70% transfected cells and lysates with no detectable Flag-MBP-1 breakdown products were used for further analysis.

For immunofluorescence assays, 1.5x10^5^ SKBr3 cells were grown onto glass coverslips in 12-well culture plates for 24 hrs, then transfected with either 750 ng of pFLAG-MBP1 or pEGFPN1 plasmid (Clontech, Mountain View, CA), as described previously [19].

For reporter assays cells (6×10^5^) were transfected with 750 ng of the pGL-cmp luciferase reporter construct and 250 ng of the β-galactosidase expressing vector pSVβ-gal (Promega, Madison, WI), the latter used as an internal control plasmid to monitor transfection efficiency. In cotransfection experiments with the pFLAG-MBP1 effector vector (1.25 μg), the total amount of DNA was kept constant by addition of the empty expression plasmid. Luciferase and beta-galactosidase activities were measured independently in duplicate using the Bright-Glo Luciferase Assay and Beta-Glo Assay Systems (Promega, Madison, WI) and a Turner 20/20 luminometer (Turner Designs, Inc., Sunnyvale, CA). Luciferase activity was normalized with respect to beta-galactosidase activity. All transfections were performed in triplicate and results from three independent experiments are expressed as mean ± SD.

### Total RNA isolation and quantitative real-time PCR

Total RNA was extracted using Trizol reagent (Invitrogen, Carlsbad, CA) according to the manufacture’s instructions. RNA was reverse-transcribed with the Superscript II reverse transcriptase (Invitrogen, Carlsbad, CA) and cDNA amplified as described previously [23] using either c-MYC or ERBB2 specific primers (Qiagen, Hilden, Germany) and Power SYBER Green PCR ready-mix in a 7300 thermal cycler (Applied Biosystems, Foster City, CA), primer sequences are listed in (Additional file 1: Table S1). PCR conditions were: denaturation at 95C° for 3 minutes, followed by 35 cycles at 95C° for 20 seconds, 60C° for 15 seconds, and 72C° for 15 seconds, and a final extension at 72°C for 7 minutes. Reaction specificity was controlled by post-amplification melting curve analysis and agarose gel electrophoresis of the amplified products. To correct for the experimental variations between samples, Ct value of TBP mRNA was determined in each PCR reaction using specific primers (Qiagen, Hilden, Germany). Data shown were generated from three independent experiments performed in triplicates and are expressed as mean ± SD. Comparison and statistical analysis were performed using Student *t* test.

### Immunofluorescence and microscopy

SKBr3 breast cancer cells were seeded onto glass coverslips in a 12-well plate culture vessel, 48–72 hrs post-transfection cells were fixed with 3.7% paraformaldehyde in phosphate buffered saline (PBS) and then permeabilized with 0.3% Triton X-100 in PBS. To detect endogenous ErbB2 and ectopically expressed Flag-MBP-1 proteins cells were incubated with 1 ug/ml of mouse anti-ErbB2 (sc-80898, Santa Cruz Biotechnology, Santa Cruz, CA) and rabbit anti-Flag (F7425, Sigma Chemical Company, St Louis, MO) primary antibodies in PBS containing 0.2% Tween 20. AlexaFluor 488-conjugated goat anti-rabbit IgG and AlexaFluor 594-conjugated goat anti-mouse IgG (Invitrogen, Carlsbad, CA) at a dilution of 1:600 were used as secondary antibodies. DNA was counterstained with 4^′^6-diamidino-2-phenylindole (DAPI) and the coverslips were mounted onto glass slides with Slowfade reagent (Invitrogen, Carlsbad, CA). Primary-antibody-omission demonstrated the specificity of the immunostaining. Immunofluorescence microscopy was performed with either a Leica DM-RA2 microscope, or a Leica TCS SP5 confocal laser-scanning microscope and confocal optical sections were created using Leica confocal software.

### Immunoblotting and immunohistochemistry

Total cell lysates from transfected cells were prepared in RIPA buffer (50 mM TrispH 7.4, 150 mM NaCl, 1% Triton X-100, 0.1% SDS, 1% sodiumdeoxycholate, 1 mM EDTA, 0.5 mM DTT) supplemented with protease and phosphatase inhibitors (Sigma Chemical Company, St Louis, MO). Frozen normal and tumor tissues were homogenized and lysates prepared as described previously [23]. Protein concentrations of tissue and cell lysates were determined by the Bradford protein assay (BioRad, Hercules, CA). Samples (30–40 ug) were separated on 4-12% polyacrylamide gradient gels (Invitrogen, Carlsbad, CA), and transferred to PVDF membrane, according to the manufacturer’s instructions (Amersham Biosciences, Sweden). Membranes were probed with primary antibodies: rabbit anti-Flag (F7425, Sigma Chemical Company, St Louis, MO, dilution 1:200), rabbit anti-ErbB2, (18299-1-AP, Proteintech, dilution 1:100), mouse anti-Myc (sc-40, Santa Cruz Biotechnology, Santa Cruz, CA, dilution 1:200) rabbit anti-HDAC1 (ab7028, Abcam, Cambridge, UK, dilution 1:500) and horseradish peroxidase-conjugated secondary antibodies (Amersham Bioscience, Sweden). Membranes were additionally probed with mouse beta-actin antibody (AC-15, Sigma Chemical Company, St Louis, MO) as a loading control. Detection was performed with a chemiluminescent substrate (Pierce Biotechnology, Rockford, IL) and signals were quantified by densitometric analysis employing the AlphaEasyFc software (Alpha Innotech Corporation, Johannesburg, South Africa).

Immunohistochemistry was performed on tissue serial sections of archived formalin-fixed, paraffin-embedded tissue blocks from patients as described previously [23], using primary antibodies against ErbB2 (4B5, Ventana Medical System, dilution 1:500), MBP-1/alpha-enolase (monoclonal antibodies ENO-19/8 and ENO-276/3, 1.0 ug/ml, [23]) and HDAC1 (ab7028, Abcam, dilution 1:1000). To confirm the specificity of immunoreactions, the primary antibody was either omitted or replaced by non-immune IgG. Tissue slides were evaluated blindly by two authors (ER and CL). The imunohistochemical grading scale used to evaluate the intensity and percentage of MBP-1-positive cells has been described previously [23]. Tumors were graded as ErbB2-positive with a score of 3+ and negative with a score of 0 or 1+, according to common pathological guidelines. Tumors ErbB2-positive 2+ were further evaluated by in situ hybridization (FISH) with a dual-color probe (PathVysion ErbB2/CEP17; Vysis, Downers Grove, IL, USA), according to manufacturer’s instructions, and scored positive when ErbB2 gene amplification was found. Immunohistochemical score for HDAC1 expression in each tissue section was calculated as the percentage of positively stained cells on total cells.

### Chromatin immunoprecipitation (ChIP) assay

In vivo MBP-1 and HDAC1 occupancy at the ERBB2 and c-MYC promoter was investigated using a ChIP assay kit (Upstate Biotech, Billerica, MA). Sheared chromatin samples from either pFlag-MBP1- or pFlag-CMV-transfected SKBr3 cells were separately immunoprecipitated with rabbit anti-Flag, anti-HDAC1 or anti-acetylated Histone H4 polyclonal antibodies (Upstate Biotech, Billerica, MA). The recovered DNA was analyzed by quantitative real-time PCR as described previously [25], using primers specific to either ERBB2 or c-MYC promoter, and to unrelated sequences as a negative control (Additional file 1: Table S1). A DNA sample representing 10% of the total input chromatin was also included as a positive control. The data shown are means ± standard deviations (SD) from three independent experiments performed in triplicates and are expressed as percentage of total input DNA.

### Statistical analysis

Group comparison and statistical analyses were performed using the software tools in GraphPad Prism version 4.02 for Windows (GraphPad Software, Inc. La Jolla, CA, USA). All tests of statistical significance were two-tailed and p-values less than 0.05 were considered statistically significant.

## Results

### MBP-1 negatively regulates ERBB2 expression

**Figure 1:**
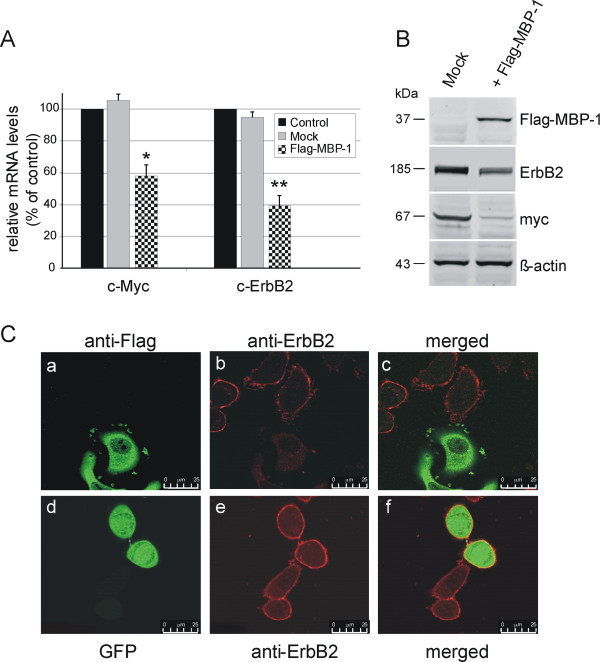
***MBP-1 negatively regulates ERBB2 and c-MYC expression in SKBr3 breast cancer cells.*** (**A**) Quantitative analysis of endogenous c-MYC and ERBB2 transcripts by qRT–PCR. SKBr3 cells were transfected with either a vector expressing MBP-1 (pFlag-MBP-1) or an empty vector (mock) and analyzed 48 hrs after transfection. Histograms show fold changes in the expression of c-MYC and c-ERBB2 mRNA after normalization with TBP. Each data point is the average of at least three independent transfection experiments, bars represent standard deviation and p values (* *P*< 0.05, ** *P*<0.005) indicate statistical significance. (**B**) Western blot analysis of myc and ErbB2 proteins in SKBr3 cells overexpressing Flag-MBP-1 and in the mock control. Both mRNA and protein levels were reduced in transfected cells. (**C**) Representative confocal microscopy images showing the intracellular localization of endogenous ErbB2 protein and either ectopically-expressed Flag-MBP-1 or GFP protein. After transfection, SkBr3 cells expressing Flag-MBP-1 were double-stained with mouse anti-ErbB2 and rabbit anti-Flag antibodies (panels a-c), GFP-expressing cells were single-stained with anti-ErbB2 primary antibodies (panels d-f). Right panels show the merged image of the middle and left panels. Scale bar, 25 um. For supplementary images, see Additional file 2.

To test the effect of MBP-1 overexpression on the endogenous ERBB2 gene, we transfected SKBr3 breast cancer cells with either a plasmid vector encoding a Flag-tagged MBP-1 protein (Flag-MBP-1) or an empty vector as a negative control; we then measured ERBB2 and c-MYC mRNA and protein expression levels by quantitative real-time PCR and Western blot, respectively (Figure 1A, B). In SKBr3 cells, which carry an amplification of the ERBB2 locus, the endogenous MBP-1 protein was barely detectable (data not shown). The overexpression of Flag-MBP-1 resulted in a significant reduction in endogenous c-MYC and ERBB2 transcript levels, 45% and 59% respectively, while no significant changes occurred after transfection with the empty vector (Figure 1A). Consistent with these results, Myc and ErbB2 protein levels were significantly reduced (Figure 1B). We then performed immunofluorescence analysis to investigate the level of the ErbB2 protein and its subcellular localization at the single cell level. As expected, a marked reduction of the ErbB2 protein along the cell membrane was observed in Flag-MBP-1-expressing cells (Figure 1C, a-c, and Additional file 2: Figure S1), whereas the level and localization of the ErbB2 protein were unchanged in SKBr3 cells transfected with the control vector expressing Green Fluorescent Protein(GFP) (Figure 1C, d-f, and Additional file 2: Figure S1).

As previously reported for the c-MYC gene, these results indicate that the exogenous MBP-1 protein negatively affects ERBB2 expression at both the mRNA and protein levels.

### MBP-1 represses the transcriptional activity of the ERBB2 promoter

**Figure 2:**
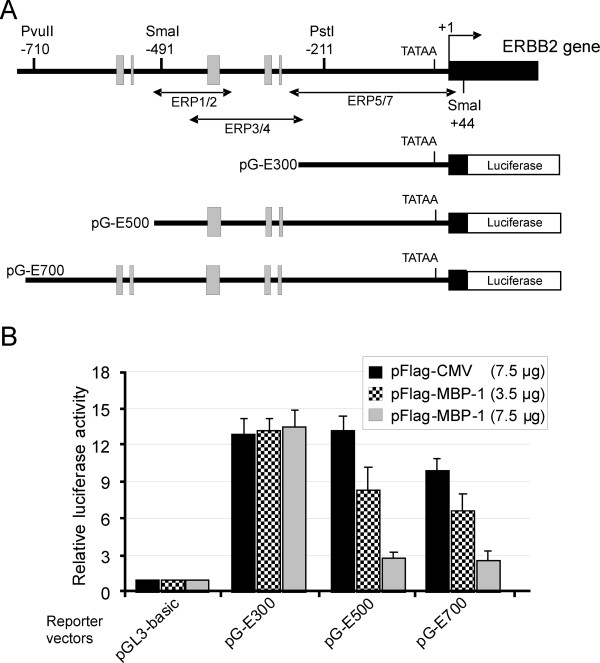
***MBP-1 represses ERBB2 promoter activity*. **(**A**) Schematic representation of ERBB2 exon-1 (black box) and 5^′^-flanking region. The TATA-box, the major transcriptional start site (+1), the position of relevant restriction sites and the location of A/T-rich sequences (gray boxes) are indicated. The numbers refer to the major transcription start site according to NCBI Ref Seq NG_007503.1. Sequences amplified by the three primer sets used in ChiP-qPCR assays are underlined. The schematic structures of the reporter plasmids, containing fragments of the human ERBB2 promoter upstream of the firefly luciferase gene, are shown below (see Additional file 1: Table S1 for details). (**B**) Functional analysis of the ERBB2 promoter in SkBr3 cells. Cells were transiently cotransfected with each reporter plasmid and two different amounts of the vector expressing Flag-MBP-1 (3.5 or 7.5 μg) or with the highest amount of the empty vector pFlag-CMV (7.5 ug). Values of luciferase activity, corrected for transfection efficiency, are expressed relative to the activity obtained with the pGL3-basic plasmid to which was assigned the value of 1. Each data point is the average of at least three independent experiments and the error bars represent SD.

To address the question of whether MBP-1 plays a regulatory role in controlling the transcription of the ERBB2 gene, the transcriptional activity of the promoter and 5^′^-flanking sequences were tested in SKBr3 cells overexpressing exogenous MBP-1. We generated deletion mutants of the human ERBB2 promoter region, extending up to 0.7 kb from the transcription start site, and inserted them in a luciferase reporter vector. The derived plasmids, named pG-E300, pG-E500 and pG-E700 (Figure 2A), were transiently cotransfected into SKBr3 cells with the effector plasmid expressing Flag-MBP-1 or with the empty pFlag-CMV vector as a negative control. As shown in Figure 2B, luciferase activity in cells cotransfected with either the pG-E500 or pG-E700 construct and Flag-MBP-1 exhibited markedly lower luciferase activities compared to cells transfected with the control vector. Furthermore, the decrease in luciferase activity was proportional to the amount of Flag-MBP-1 plasmid transfected.

Activity of the pG-E300 reporter plasmid, which was 10−13 times greater than the activity obtained in the presence of the promoterless construct pGL3-basic, was unaffected by MPB-1 expression.

These results indicate that the region between nucleotide −514 and −262 of the ERBB2 proximal promoter contains cis-acting sequences responsible for the transcriptional repression exerted by MBP-1. Indeed, nucleotide sequence analysis of the ERBB2 promoter revealed the presence of several A/T-rich elements that may function as putative binding sites for MBP-1 [7]. Three of these are located between nucleotide −514 and −262 (Figure 2A and Additional file 3: Figure S2).

### MBP-1 binds to the ERBB2 promoter in vivo

**Figure 3:**
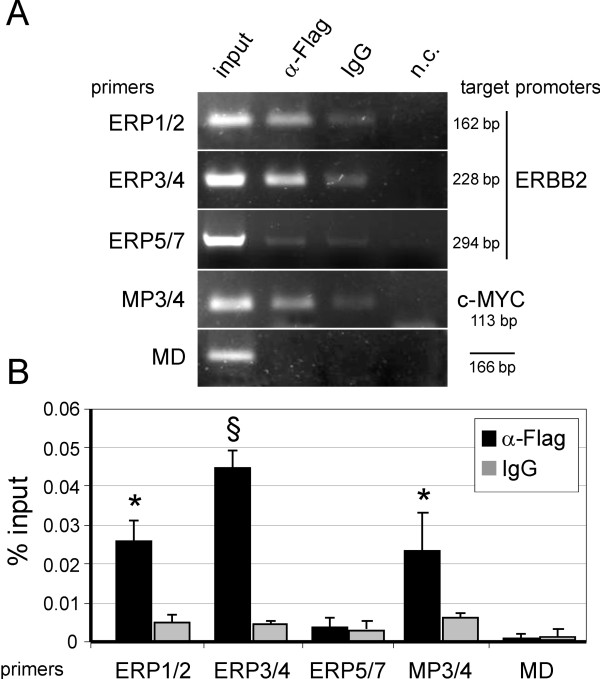
***MBP-1 interacts in vivo with ERBB2 and c-MYC promoters*. **(**A**) Identification of in vivo binding regions for MBP-1. DNA of input and immunoprecipitated chromatin samples was amplified using primers directed to the ERBB2 promoter region (ERP1/2, ERP2/3 and ERP5/7); primers targeted to the c-MYC P2 promoter (MP3/4) as a positive control; and primers directed to an unrelated region of the c-MYC gene (MD). Numbers indicate the length of the amplified DNA fragments. Reactions in absence of input DNA were included as negative controls (n.c.). (**B**) Quantification of immunoprecipitated chromatin by real-time PCR. The amount of immunoprecipitated DNA was calculated relative to that present in total input chromatin (% input). Gene-specific PCR detected in vivo binding of MBP-1 to both ERBB2 and c-MYC promoters. Each data point is the average of triplicates from three independent ChIP experiments ± SD and p values (* *P*< 0.05, § *P*<0.01) indicate statistical significance.

The results of the functional reporter analysis and in silico observations prompted us to further investigate putative interactions between cis-regulatory elements in the ERBB2 promoter and the MBP-1 protein using in vivo chromatin-immunoprecipitation (ChIP). Sheared chromatin from Flag-MBP-1-expressing SKBR3 cells was immunoprecipitated either with anti-Flag antibodies or unrelated IgG as a negative control. Genomic DNA was analyzed by PCR using three oligonucleotide pairs (ERP1/2, ERP3/4 and ERP5/7) that amplify three overlapping fragments spanning a 564-bp region from nucleotide −520 to +44 of the ERBB2 promoter (see Additional file 3: Figure S2 and Additional file 1: Table S1 for details, respectively). As positive and negative controls, we used primers directed at the c-MYC P2 promoter region (MP3/4) containing the TATA-box, a known binding site of MBP-1 [7,9], and a primer set targeted at an unrelated region of the c-MYC gene (MD) (see Additional file 1: Table S1). As shown in Figure 3A, the anti-Flag-immunoprecipitated chromatin yielded c-MYC-specific as well as ERBB2-specific PCR products (ERP1/2 and ERP3/4 primers). No enrichment was observed with ERP5/7 primers which amplify the ERBB2 promoter region containing the TATA-box, which supports the lack of MBP-1-mediated repression we observed with the pG-300 luciferase reporter plasmid (see Figure 2B).

To further confirm specificity and to gain quantitative information about the DNA fold-enrichment in the immunoprecitated samples, we performed real-time PCR analysis. As shown in Figure 3B, ERBB2 and c-MYC genomic DNA were significantly enriched in anti-Flag precipitated samples compared to the IgG controls, at least 0.02% with respect to the ChIP input DNA. ERBB2-specific primer sets ERP1/2 and ERP3/4 gave a statistically significant enrichment; however, the pair amplifying the larger fragment (ERP3/4) yielded a greater percentage, suggesting the presence of more than one functional site for MBP-1 in the target region or, alternatively, a more efficient amplification.

### In vivo recruitment of HDAC1 to the ERBB2 promoter

**Figure 4:**
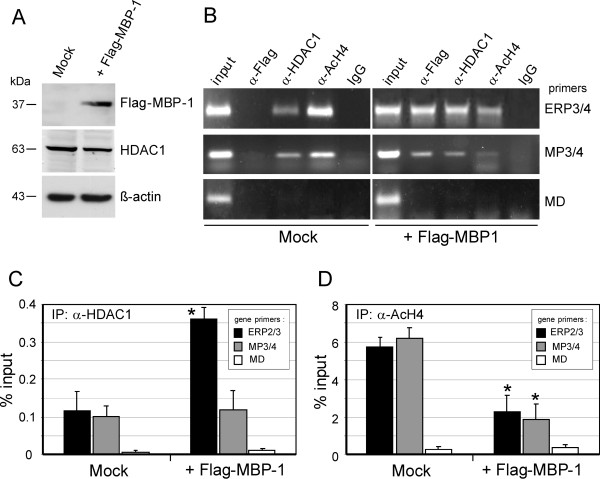
***In vivo recruitment of MBP-1 and HDAC1 proteins to ERBB2 and c-MYC promoters*. **(**A**) Immunoblot analysis of SKBr3 cells transfected with pFlag-MBP-1 or mock-transfected using anti-Flag, anti-HDAC1 and anti-beta-actin antibodies. (**B**) MBP-1 and HDAC1 occupancy at ERBB2 and c-MYC promoter DNA of input and immunoprecipitated chromatin samples was amplified using primers directed to ERBB2 promoter (ERP2/3), to c-MYC. P2 promoter (MP3/4) and primers directed to an unrelated region of the c-MYC gene (MD). (**C**, **D**) Quantification by real-time PCR of chromatin immunoprecipitated with anti-HDAC1 and anti-AcH4 antibodies. The amount of immunoprecipitated DNA was calculated relative to that present in total input chromatin (% input). Each data point is the average of triplicates from three independent ChIP experiments ± SD, p value (* *P*<0.01) indicate statistical significance.

In light of the previously described interaction of HDAC1 with MBP-1 [18], we investigated the in vivo recruitment of both proteins to ERBB2 and c-MYC promoters. As a control, lysates of mock- and pFlag-MBP-1-transfected SKBr3 cell were analyzed by Western blot to monitor the relative expression of exogenous MBP-1 and endogenous HDAC1 protein. No significant variation in the HDAC1 protein level was observed in the presence of exogenous MBP-1 (Figure 4A).

Chromatin from Flag-MBP-1-expressing SKBR3 cells and mock control was immunoprecipitated with either anti-Flag or anti-HDAC1 antibodies, and genomic DNA was analyzed using specific oligonucleotide pairs. As shown in Figure 4B, both antibodies yielded ERBB2-specific and c-MYC-specific PCR products; however, quantitative PCR analysis of HDAC-1-immunoprecipitated chromatin indicated a much greater enrichment of ERBB2 than the c-MYC promoter sequences in the presence of exogenous Flag-MBP-1 (Figure 4C).

As a further control, chromatin was also immunoprecipitated with anti-acetylated histone H4 (AcH4) antibodies. AcH4 is considered a hallmark of active transcription [26]. As expected for the negative role of MBP-1 on transcription, the AcH4-enriched chromatin samples from Flag-MBP-1-transfected cells yielded about 3 times less ERBB2 and c-MYC promoter sequences compared to mock-transfected cells (Figure 4D).

Taken together, these results demonstrate that MBP-1 binds to both ERBB2 and c-MYC promoters in vivo and indicate a possible involvement of HDAC1 in the transcriptional repression of the ERBB2 gene; in addition, our data support previous observations suggesting that MBP-1-mediated repression of the c-MYC promoter may involve the interplay of other specific cofactors besides HDAC1 [18].

### HDAC1 and MBP-1 expression in breast IDC

**Figure 5:**
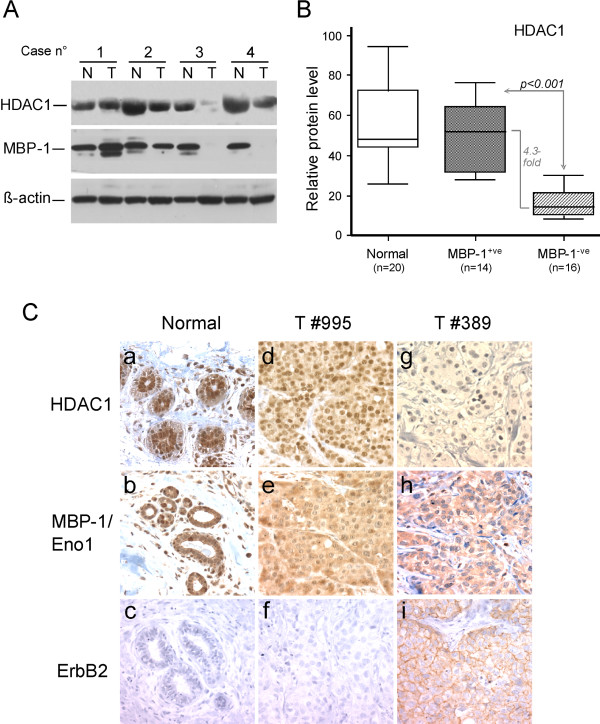
***Expression of MBP-1 and HDAC1 in primary breast tumors and adjacent normal tissues*. **(**A**) Representative Western blot analysis of HDAC1, MBP-1 and beta-actin proteins in breast tumors (T) and paired normal tissues (N). (**B**) HDAC1 protein expression levels in normal versus breast cancer tissues. Proteins were analyzed by immunoblotting and data normalized with respect to beta-actin. The Box plot represents the HDAC1/beta-actin ratio determined in 20 normal tissue (normal), 14 MBP-1-positive (+ve) and 16 MBP-1-negative (−ve) breast tumors. HDAC1 protein levels were associated with MBP-1 status, with a statistically significant enrichment in MBP-1-positive IDCs (4.3 fold, p<0.001). Bars above and below the boxes represent the maximum and minimum expression. Each box delineates the first to third quartiles of expression, and the central bar represents the median. (**C**) Representative immunohistochemical staining of normal mammary tissue (a-c), MBP-1-positive (#995) and MBP-1-negative (#389) IDC tumors (d-f and g-i, respectively). HDAC1 and MBP-1 nuclear staining in normal tissues (panels a, b) and tumors (panels d, e) correlated with undetectable ErbB2 expression (panels c, f). Magnification: 300x.

The results reported above on the functional role exerted by MBP-1 in the negative transcriptional control of ERBB2 promoter support the inverse correlation we previously found between MBP-1 and ErbB2 expression levels in primary breast tumors [23]. Moreover, the potential involvement of HDAC1 in the ERBB2 promoter transcriptional repression is in agreement with previous studies associating HDAC1 expression with breast cancer progression and survival [27-29]. Seeking new insights, we analyzed MBP-1 and HDAC1 protein levels in total lysates from a set of primary IDCs and the paired normal breast tissue samples. A representative immunoblot analysis is shown in Figure 5A. Most of the normal breast tissue showed a concomitant higher expression of MBP-1 and HDAC1 than the paired tumor samples. Comparison of HDAC1 expression in normal tissues (n=20) and in MBP-1-positive (n=14) and MBP-1 negative (n=16) primary IDCs indicated that HDAC1 expression was significantly higher in MBP-1-positive compared to MBP-1-negative tumors (4.3 fold, p=0.001), whereas no significant difference was observed between MBP-1-positive tumors and normal tissues (Figure 5B). Given that no significant variation in the HDAC1 protein level was observed in SkBr3 cells transiently overexpressing MBP-1 (Figure 4A), we may exclude a direct positive role of MBP-1 on HDAC1 protein expression and/or stabilization.

**Figure 6:**
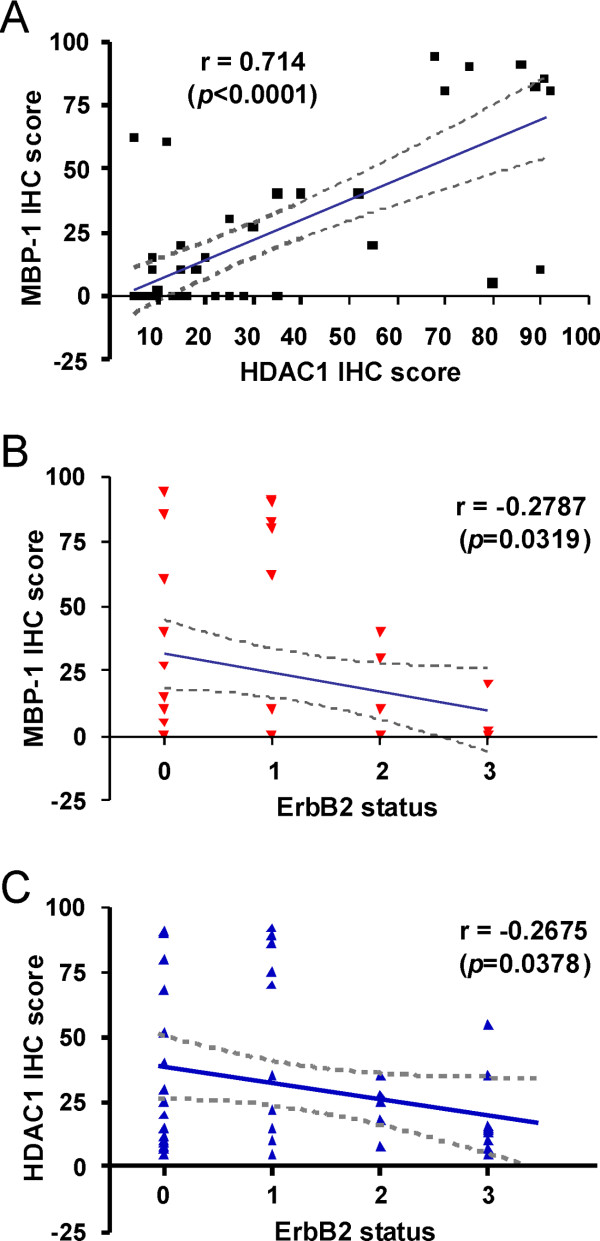
***Correlations between MBP-1, HDAC1 and ErbB2 protein expression in primary breast tumors.*** Correlation plot for MBP-1 versus HDAC1 (**A**), MBP-1 versus ErbB2 (**B**) and HDAC1 versus ErbB2 (**C**) protein levels. Black squares and coloured triangles represent expression values determined by immunohistochemical staining of 45 breast IDCs, as described in Materials and Methods. Blue lines represent the linear regression, dotted lines the 95% CI. The coefficient of correlation (r) was determined and its statistical significance was tested using the nonparametric Spearman rank correlation test.

To further investigate MBP-1 and HDAC1 expression and localization and to correlate their expression to ErbB2 status, we analyzed a total of 45 primary IDCs by immunohistochemistry (IHC), including the adjacent normal breast tissue in almost every case (41 out of 45). As expected, HDAC1 gave a nuclear staining in all the samples, whereas MBP-1 nuclear staining was observed in almost all the normal tissue but in only 22/45 tumors (48%). Figure 5C shows a representative MBP-1, HDAC1 and ErbB2 serial section staining of normal tissue, MBP-1-positive (T #995) and MBP-1-negative (T #389) tumors. In summary, strong HDAC1 and MBP-1 nuclear staining was observed in normal tissues (panels a, b); strong to moderate HDAC1 and MBP-1 nuclear reactivity was detected in ErbB2-negative tumors (panels d, e, f), whereas low nuclear staining was detected in ErbB2-positive tumors (panels g, h, i). These IHC results are in agreement with what was observed by Western blot (Figure 5B), and statistical data analysis indicated the existence of a highly significant correlation between MBP-1 and HDAC1 expression in tumors (p<0.0001), with a Spearman rank correlation coefficient equal to 0.714 (Figure 6A). On the other hand, ERBB2 expression negatively correlated with both MBP-1 and HDAC1 protein expression (p = 0.031 for MBP-1 and 0.037 for HDAC1), with Spearman rank correlation coefficients equal to −0.278 and −0.267, respectively (Figure 6B, C). Although these negative correlations with ErbB2 status are weak, likely because of the limited number of samples, they still support the hypothesis of a negative regulatory network linking MBP-1 and HDAC1 to ERBB2 expression in breast IDC.

## Discussion

In this study, we provide novel observations regarding the transcriptional control of the ERBB2 gene in SKBr3 breast cancer cells. The human ERBB2 gene is frequently amplified in breast tumors, and its high expression is associated with poor prognosis. However, substantial evidence suggests that the increased level of ERBB2 mRNA depends on active gene transcription in addition to gene amplification [30]. Several positive and negative regulatory elements have been characterized in the ERBB2 gene proximal promoter as well as in the 5^′^-flanking sequence up to 6 kb and in the first intron [31-34]. Altogether, these studies indicate the involvement of several factors regulating ERBB2 gene transcription in breast cancer cells. Among positive regulators, members of the AP-2 and Ets families of transcription factors are required for maximal ERBB2 promoter activity and have been associated with the overexpression of the gene in breast cancer (for a review, see [35]); in addition, the multifunctional transcription factor YY1 has been shown to cooperate with AP-2 to stimulate ERBB2 promoter activity through the AP-2 binding sites [36]. Other transcription factors have been identified as negative regulators of ERBB2 expression in breast cancer (for a review, see [37]): e.g., PEA3, an Ets DNA-binding protein that targets a DNA motif in the ERBB2 gene promoter [38]; FOXP3, an X-linked breast cancer tumor suppressor which represses the transcription of the ERBB2 gene by interacting with forkhead DNA-binding motifs in the promoter [39]; the zinc-finger transcription factor GATA4, part of a negative feedback regulatory loop with the ERBB2 gene [40]. Although the functional relationships between positive and negative transacting factors still remain largely unexplored, overall, these data illustrate the complexity of ERBB2 gene transcriptional control.

In this context, we report that the c-MYC gene repressor MBP-1 negatively regulates ERBB2 gene transcription in SKBr3 breast cancer cells by targeting regulatory sequences in the promoter region. Through chromatin immunoprecipitation, we have located the MBP-1 binding region between nucleotide −514 and −262, relative to the major transcriptional start site of the ERBB2 gene, and demonstrated the concomitant recruitment of HDAC1 to the same region. Furthermore, our ChIP assays have indicated a decreased AcH4 occupancy at the same ERBB2 promoter region targeted by MBP-1 and HDAC1, suggesting a regulatory role for HDAC1 in MBP-1 repression activity, although we cannot exclude the involvement of other HDAC family members.

HDAC1 has positive and negative effects on gene transcription [41] and, like all the HDACs, lacks a DNA-binding domain; thus, it must be associated with a DNA-binding protein in order to target a specific chromatin region (reviewed in [42]). For example, to repress transcription, HDAC1 interacts with the transcription factor E2F in a complex containing BRM, BRG1, and SUV39H1 [43]. Ghosh et al. previously demonstrated that MBP-1 physically associates with HDAC1 in vitro and in vivo, although the MBP-1-mediated repression of the c-MYC P2 promoter seems to be independent of HDAC1 [18]. Our results support this previous observation concerning the c-MYC promoter and, conversely, suggest that MBP-1 represses ERBB2 gene transcription by recruiting the HDAC1 protein to its promoter. Therefore, MBP-1-mediated transcriptional repression may occur through different mechanisms, likely depending on the chromatin structure and the nucleotide sequence of the promoter. MBP-1 can block the assembly of the basal transcription complex by competing with TBP, as reported for the c-MYC P2 promoter [7], or it may bind the promoter regulatory sequences and recruit HDAC1, as we suggest here, for the ERBB2 gene. The differences we observed in the recruitment of HDAC1 to ERBB2 and the c-MYC P2 promoter strongly support this last hypothesis.

Overall, our data suggest the existence of a novel transcriptional regulatory network that modulates ERBB2 expression, though detailed investigations using different cellular models are needed to dissect this network and define the molecular mechanisms underlying MBP-1/HDAC1-mediated transcriptional repression of the ERBB2 gene in breast cancer.

We also report a significant inverse correlation between ERBB2 expression and both MBP-1 (r= −0.278, p= 0.031) and HDAC1 (r= −0.267, p= 0.037) protein levels in primary breast tumors, and, accordingly, we propose MBP-1/HDAC1/ERBB2 relative expression as a diagnostic marker in breast IDC. Our results are in agreement with previous observations that have associated the reduction of HDAC1 transcript and protein levels with progression from normal mammary epithelium to ductal carcinoma in situ (DCIS) and to IDC [27-29].

Furthermore, it has been independently reported that the expression of either MBP-1 or HDAC1 is a predictor of good disease-free survival, and both proteins are independent prognostic factors in breast cancer patients [23,29]. Despite the limited number of patients examined in this study, the significant positive correlation we observed between MBP-1 and HDAC1 expression in ErbB2-negative IDC suggests that their concomitant high expression may have a stronger diagnostic and prognostic significance in this tumor subtype.

## Conclusions

In summary, we have identified ERBB2 as a novel target gene of MBP-1. We demonstrate that MBP-1 negatively controls ERBB2 expression in SKBr3 breast cancer cells and suggest a role for HDAC1 in this regulatory mechanism. We show for the first time that a concomitant high expression of MBP-1 and HDAC1 inversely correlates with ERBB2 expression in primary breast tumors.

The data presented here provide the basis for future studies involving a larger number of patients with a long follow-up period to further elucidate the functional and prognostic relevance of MBP-1 and HDAC1 in breast cancer.

## Competing interests

The authors declare that they have no competing interests.

## Authors’ contributions

Conceived and designed the experiments: FC CM ER AG SF. Performed the experiments: FC CM AF MLP CL GP. Analyzed the data: FC CM ER AG SF. Contributed reagents/materials/analysis tools: FC CM CL GP AG SF. Wrote the paper: FC AG SF. All authors read and approved the final manuscript.

## Authors’ information

Agata Giallongo and Salvatore Feo share senior co-authorship.

## Pre-publication history

The pre-publication history for this paper can be accessed here:

http://www.biomedcentral.com/1471-2407/13/81/prepub

## Supplementary Material

Additional file 1: Table S1 List of gene-specific oligonucleotides used in this study. Click here for file

Additional file 2: Figure S1 *Immunofluorescence microscopy images showing intracellular localization of endogenous ErbB2 and either ectopically expressed MBP-1 or GFP protein. *Human SKBr3 cancer cells transiently expressing either Flag-MBP-1 or GFP protein (upper and lower panels, respectively) were fixed, permeabilized and double-stained with anti-ErBB2 and anti-Flag antibody or single-stained with anti-ErbB2, as indicated. Nuclei were stained with DAPI. Spatial distribution was visualized by light microscopy as described in Materials and Methods. The colour merged images show the loss of ErbB2 membrane staining in MBP-1-expressing cells (upper panel). Scale bar, 25 um. Click here for file

Additional file 3: Figure S2 *Nucleotide sequence of the human ERBB2 promoter and upstream regions. *The nucleotide sequence is numbered with the major transcription start site designated as + 1 (according to NCBI RefSeq: NG_007503.1). Positions of relevant restriction sites are indicated and A/T-rich elements are boxed. Arrows indicate the position of oligonucleotides used for the construction of the ERBB2-luciferase reporter plasmids and for ChIP-qPCR assays (see Additional file 1). Click here for file
