# Supplementary material for: Negative transcriptional control of ERBB2 gene by MBP-1 and HDAC1: diagnostic implications in breast cancer
Source: BMC Cancer. 2013 Feb 19;13:81. doi: 10.1186/1471-2407-13-81 (PMC3599235; doi:10.1186/1471-2407-13-81)
Supplement: Additional file 1: Table S1 — List of gene-specific oligonucleotides used in this study. [file 1471-2407-13-81-S1.pdf]

Contino et al., supplementary table S1

Table S1. List of gene-specific oligonucleotides used in this study

(**A**) Oligonucleotides used for ERBB2 promoter luciferase reporter construct

Target sites*	Sense (S) and Antisense (A) sequences (5’ to 3’)§	Amplicon size (bp)
HER-5	S: GCTAGCGCTGGTCATGGTGGCACA	787
HER-6	S: GCTAGCACTTCAAAGATTCCAGAAGATATGC	558
HER-7	S: GCTAGCCACCAGCCTCTGCATTTAGG	306
HER-3	A: GAAGATCTGGGCTCCCCTGGTTTCTC	-

(**B**) Real time PCR oligonucleotides for ChIP assay

Target genes	Forward (F) and reverse (R) sequences (5’ to 3’)	Amplicon size (bp)
ERBB2	ERP1-F: ACTTCAAAGATTCCAGAAGATATGC<br>ERP2-R: GCTTGCATCCTACTCCATCC	162
ERBB2	ERP3-F: ACACATCCCCCTCCTTGACT<br>ERP4-R: CGGAGAATCCCTAAATGCAG	228
ERBB2	ERP5-F: CTCTGCATTTAGGATTCTCCG<br>ERP7-R: GGGCTCCCCTGGTTTCTC	294
MYC	MP3-F: AGGGCTTCTCAGAGGCTTG<br>MP4-R: TGCCTCTCGCTGGAATTACT	113
MYC	MD-F: ATT GTC CCC TCT CCT CCT GT<br>MD-R: CTT CGT CTC CCC TAC TGC TG	166

Oligonucleotide positions in the ERBB2 promoter sequence are shown in supplementary Figure 1S. § The underlined sequence refers to the NheI or BglII restriction site added to each oligonucleotide to clone the amplified fragment in the pGL3 reporter plasmid.
