# Supplementary material for: Negative transcriptional control of ERBB2 gene by MBP-1 and HDAC1: diagnostic implications in breast cancer
Source: BMC Cancer. 2013 Feb 19;13:81. doi: 10.1186/1471-2407-13-81 (PMC3599235; doi:10.1186/1471-2407-13-81)
Supplement: Additional file 2: Figure S1 — Immunofluorescence microscopy images showing intracellular localization of endogenous ErbB2 and either ectopically expressed MBP-1 or GFP protein. Human SKBr3 cancer cells transiently expressing either Flag-MBP-1 or GFP protein (upper and lower panels, respectively) were fixed, permeabilized and double-stained with anti-ErBB2 and anti-Flag antibody or single-stained with anti-ErbB2, as indicated. Nuclei were stained with DAPI. Spatial distribution was visualized by light microscopy as described in Materials and Methods. The colour merged images show the loss of ErbB2 membrane staining in MBP-1-expressing cells (upper panel). Scale bar, 25 um. [file 1471-2407-13-81-S2.pdf]

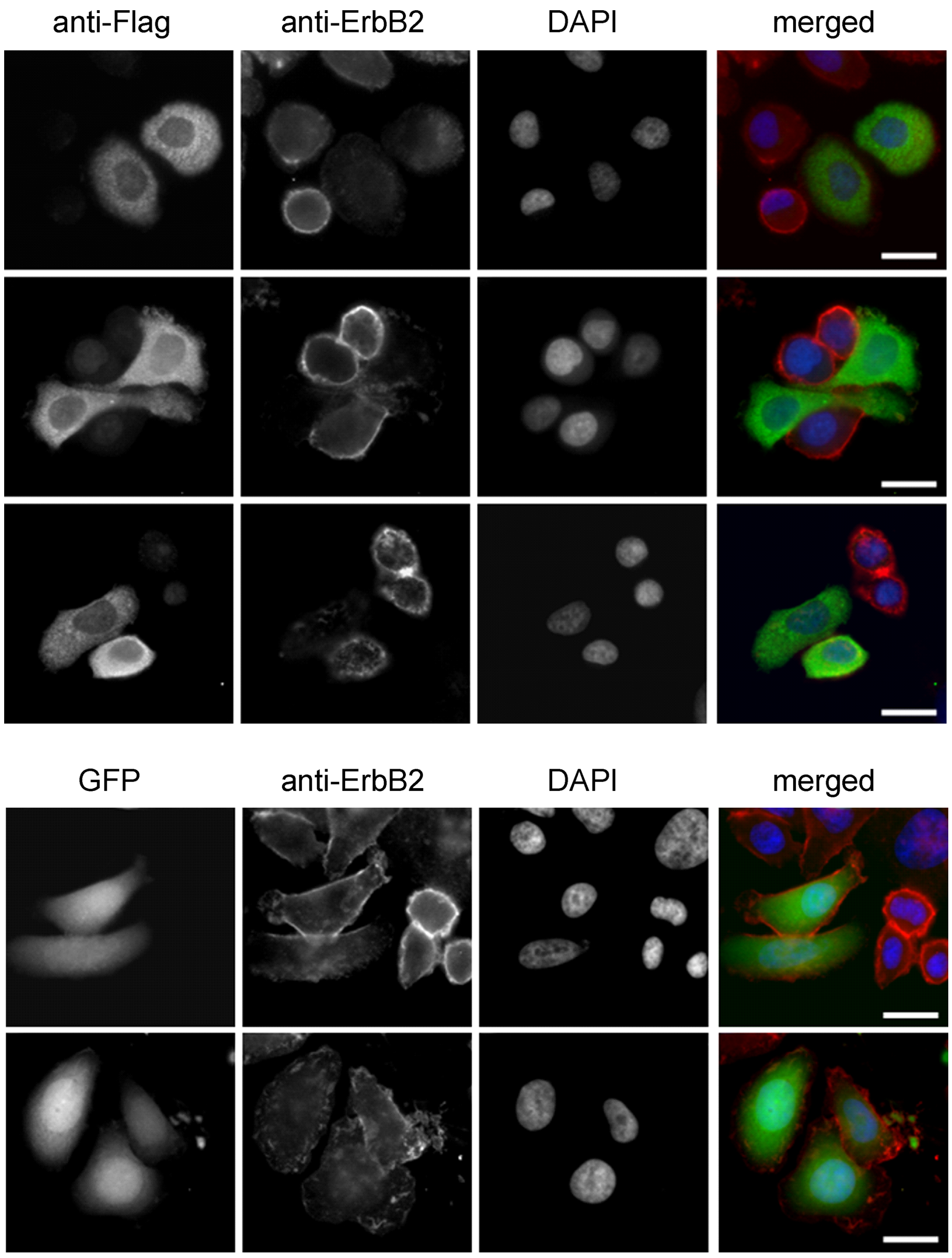

Figure S1. *Immunofluorescence microscopy images showing intracellular localization of endogenous ErbB2 and either ectopically expressed MBP-1 or GFP protein.* Human SKBr3 cancer cells transiently expressing either Flag-MBP-1 or GFP protein (upper and lower panels, respectively) were fixed, permeabilized and double-stained with anti-ErBB2 and anti-Flag antibody or single-stained with anti-ErbB2, as indicated. Nuclei were stained with DAPI. Spatial distribution was visualized by light microscopy as described in Materials and Methods. The colour merged images show the loss of ErbB2 membrane staining in MBP-1-expressing cells (upper panel). Scale bar, 25 um.
