# Supplementary material for: Negative transcriptional control of ERBB2 gene by MBP-1 and HDAC1: diagnostic implications in breast cancer
Source: BMC Cancer. 2013 Feb 19;13:81. doi: 10.1186/1471-2407-13-81 (PMC3599235; doi:10.1186/1471-2407-13-81)
Supplement: Additional file 3: Figure S2 — Nucleotide sequence of the human ERBB2 promoter and upstream regions. The nucleotide sequence is numbered with the major transcription start site designated as + 1 (according to NCBI RefSeq: NG_007503.1). Positions of relevant restriction sites are indicated and A/T-rich elements are boxed. Arrows indicate the position of oligonucleotides used for the construction of the ERBB2-luciferase reporter plasmids and for ChIP-qPCR assays (see Additional file 1). [file 1471-2407-13-81-S3.pdf]

Contino et al., supplementary figure S2

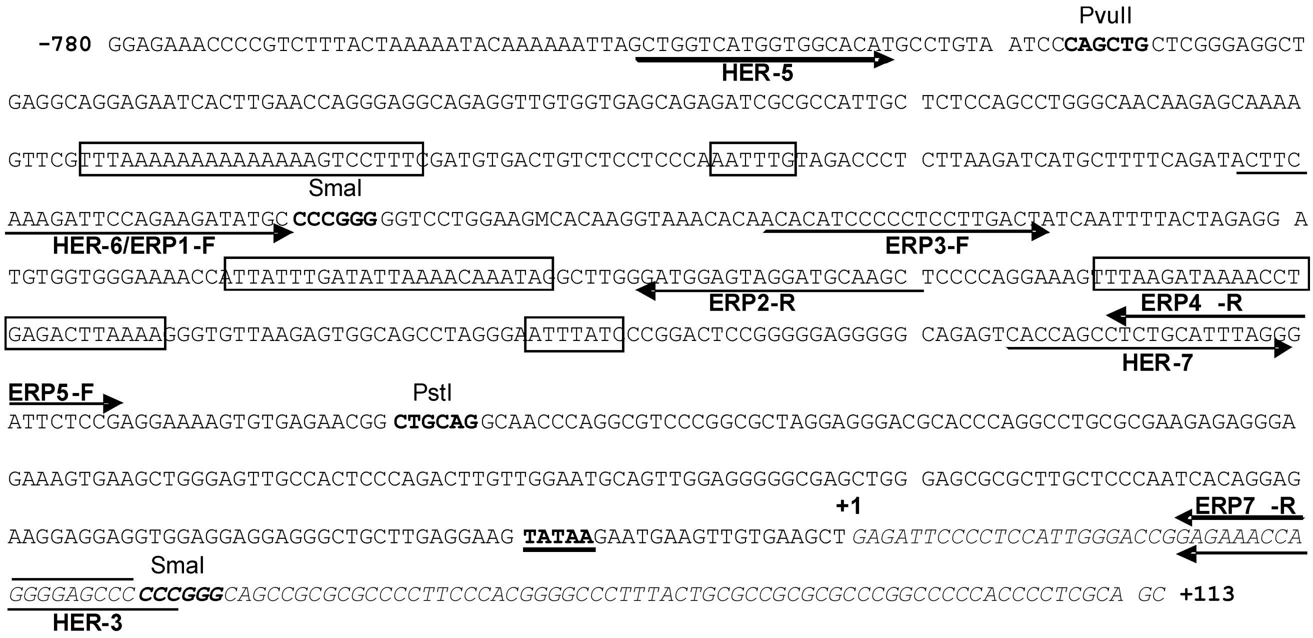

Figure S2. *Nucleotide sequence of the human ERBB2 promoter and upstream regions.* The nucleotide sequence is numbered with the major transcription start site designated as + 1 (according to NCBI RefSeq: NG_007503.1). Positions of relevant restriction sites are indicated and A/T-rich elements are boxed. Arrows indicate the position of oligonucleotides used for the construction of the ERBB2-luciferase reporter plasmids and for ChIP-qPCR assays (see additional_file_1).
